# Does the Axillary Lymph Node Ratio Have Any Added Prognostic Value over pN Staging for South East Asian Breast Cancer Patients?

**DOI:** 10.1371/journal.pone.0045809

**Published:** 2012-09-24

**Authors:** Nakul Saxena, Mikael Hartman, Cheng-Har Yip, Nirmala Bhoo-Pathy, Lay Wai Khin, Nur Aishah Taib, Lai-Meng Looi, Siew-Eng Lim, Soo-Chin Lee, Helena M. Verkooijen

**Affiliations:** 1 Saw Swee Hock School of Public Health, National University of Singapore, Singapore, Singapore; 2 Department of Surgery, Yong Loo Lin School of Medicine, National University of Singapore, Singapore, Singapore; 3 Department of Surgery, Faculty of Medicine, University of Malaya, Lembah Pantai, Kuala Lumpur, Malaysia; 4 National Clinical Research Centre, Level 3, Dermatology Block, Hospital Kuala Lumpur, Jalan Pahang, Kuala Lumpur, Malaysia; 5 Investigational Medicine Unit, National University Health Systems, National University of Singapore, Singapore, Singapore; 6 Department of Pathology, Faculty of Medicine, University of Malaya, Lembah Pantai, Kuala Lumpur, Malaysia; 7 Department of Hematology Oncology, National University Cancer Institute, National University Health System, Singapore, Singapore, Singapore; 8 Imaging Division, University Medical Center Utrecht, Utrecht, The Netherlands; University Medical Centre Utrecht, The Netherlands

## Abstract

**Introduction:**

Lymph node ratio (LNR, i.e. the ratio of the number of positive nodes to the total number of nodes excised) is reported to be superior to the absolute number of nodes involved (pN stage) in classifying patients at high versus low risk of death following breast cancer. The added prognostic value of LNR over pN in addition to other prognostic factors has never been assessed.

**Methods:**

All patients diagnosed with lymph node positive, non-metastatic invasive breast cancer at the National University Hospital (Singapore) and University of Malaya Medical Center (Kuala Lumpur) between 1990–2007 were included (n = 1589). Overall survival of the patients was estimated by the Kaplan Meier method for LNR [categorized as low (>0 and <0.2), intermediate (0.2–0.65) and high (>0.65–1)] and pN staging [pN1, pN2 and pN3]. Adjusted overall relative mortality risks associated with LNR and pN were calculated by Cox regression. The added prognostic value of LNR over pN was evaluated by comparing the discriminating capacity (as indicated by the c statistic) of two multivariate models, one including pN and one including LNR.

**Results:**

LNR was superior to pN in categorizing mortality risks for women ≥60 years, those with ER negative or grade 3 tumors. In combination with other factors (i.e. age, treatment, grade, tumor size and receptor status), substituting pN by LNR did not result in better discrimination of women at high versus low risk of death, neither for the entire cohort (c statistic 0.72 [0.70–0.75] and 0.73 [0.71–0.76] respectively for pN versus LNR), nor for the subgroups mentioned above.

**Conclusion:**

In combination with other prognosticators, substitution of pN by LNR did not provide any added prognostic value for South East Asian breast cancer patients.

## Introduction

Axillary lymph node status is one of the most important prognostic factors for breast cancer [1], [2], [3]. Traditionally, axillary lymph node status is classified according to the American Joint Committee on Cancer (AJCC) breast cancer staging system, which is based on the number of positive axillary lymph nodes [4] where pN0 indicates zero positive nodes, pN1 1–3 positive nodes, pN2 4–9 positive nodes and pN3≥10 positive nodes. This pN stage is restricted by the number of nodes excised [5] which in turn depends upon the surgical approach to axillary dissection, the expertise of the surgeon as well as the pathologists’ experience and thoroughness. Variation in these factors can lead to large differences in the number of lymph nodes retrieved across institutions thereby influencing staging.

Increasing evidence suggests that the Lymph Node Ratio (LNR) (the ratio of the number of positive nodes to the total number of nodes excised), is a superior prognostic indicator compared to the absolute number of nodes involved [6], [7], [8], [9], [10]. However some studies have shown no difference in prognostic value for LNR over pN [11]. Vinh Hung *et al* showed that LNR, categorized as low >0 and <0.2, intermediate 0.2 to 0.65 and high risk >0.65 to 1, was better at predicting breast cancer specific mortality than pN staging [6]. This conclusion was based on the fact that confidence intervals for the adjusted hazard ratios did not overlap for the intermediate and high category LNR groups but did so for the pN2 and pN3 groups. A study from Korea showed no overall difference between LNR and pN staging in categorizing poor, intermediate and good survivors, except for certain subgroups, i.e. women aged <35 years, HER2 over expressing and triple negative tumors [10]. Other studies conducted in different populations also suggested that LNR was a significant and independent predictor of outcome for breast cancer patients [7], [8], [9], [12], [13].

Prognostication, however, is a multivariable process, as the outcome of a disease is determined by a variety of (sometimes interacting) factors, and breast cancer is no exception. In addition to axillary lymph node status, prognosis is determined by a variety of factors, including, age, tumor size, grade, receptors status and treatment. Despite the large number of studies that have addressed LNR, not one has assessed the added prognostic value of LNR over pN in predicting overall survival after breast cancer. Via this study we aim to assess the added prognostic value of LNR over pN staging in the South East Asian setting by comparing the pN and LNR prediction models in terms of (1) predictive power, (2) discrimination and (3) net reclassification improvement of patient into appropriate risk categories of all cause mortality.

## Methods

Data for this study were obtained from the Singapore Malaysia Hospital-based Breast Cancer Registry [14]. This registry combines data from the National University Hospital (NUH) breast cancer registry, Singapore and the University of Malaya Medical Center (UMMC) breast cancer registry, Kuala Lumpur, Malaysia.

The NUH breast cancer registry started in 1995 and contains information on 2,449 consecutive breast cancer patients diagnosed between 1990 and 2007. The UMMC breast cancer registry started in 1993 contains information on 3,320 patients diagnosed between 1993 and 2007. Details on both these registries are described elsewhere [14], [15]. In both centers, patients were monitored through follow-up in the specialist outpatient clinics. Data on mortality were obtained from the hospitals’ medical records and by linkage with the respective death registries. Follow up for each patient was calculated from the date of diagnosis to the date of death or end of follow up (July 2010 for NUH patients and November 2010 for UMMC patients). Both the registries had approval from their respective ethics review boards.

We selected women diagnosed with non metastatic primary invasive breast cancer, with information on the number of excised and the number of positive axillary lymph nodes. Patients receiving neoadjuvant chemotherapy (N = 312), patients with a node negative (pN0) axilla (N = 2352), patients with missing information on exact number of lymph nodes involved (N = 664), with *in situ* breast cancer (N = 317) and stage IV disease (N = 535) were excluded. In total 1589 patients were included for analysis.

Information recorded for each patient included age at diagnosis, ethnicity (Chinese, Malay, Indian or others), year of diagnosis, place of diagnosis (Singapore, Kuala Lumpur), date of death or date of last contact. Tumor characteristics included tumor size (<2 cm, 2–5 cm, >5 cm, unknown), estrogen (ER) and progesterone receptor (PR) status (positive i.e., ≥10% of epithelial tumor cells expressing receptors, negative and unknown), grade (good, moderate, poor, unknown). In terms of axillary dissection, we collected information on total number of axillary nodes examined and number of positive axilary nodes. LNR was categorized into three categories including, low (>0 and <0.2), intermediate (0.2 to 0.65) and high category (>0.65 to 1) groups as previously reported [6] corresponding to low, intermediate and high risk of death respectively.

## Statistical Analysis

### Prediction Models

Life table analysis was performed to calculate survival probabilities for the three pN categories and the three LNR categories. After testing for proportionality, we performed univariate Cox proportional hazard analysis to identify variables that were significantly associated with all cause mortality. Multivariate Cox proportional hazard analysis was applied 1) to calculate adjusted mortality risks and 2) to identify which combination of factors best predicted overall survival. For this we entered all variables univariately associated with overall survival with a p-value <0.2 into the model and used stepwise backward regression and maximum likelihood method to find the optimal fit. Internal validation of each model was done by bootstrap resampling.

Two models (A and B) were constructed. Each model contained the same baseline variables, i.e., age, radiotherapy, surgery type, grade and tumor size (base model). Model A contained pN stage in addition to the base model variables while Model B contained LNR in addition to the base model variables. From the final models, adjusted Hazard Ratio for pN and LNR were derived.

Base model : age, radiotherapy, surgery type, grade and tumor size.

Model A: age, radiotherapy, surgery type, grade, tumor size and pN stage.

Model B: age, radiotherapy, surgery type, grade, tumor size and LNR.

### Discrimination and Caliberation of Prediction Models

In order to ascertain the added prognostic value of LNR over pN, we compared the discriminative capacity of model A with model B. Discrimination indicates how well the model is able to distinguish between patients who will experience the outcome (death) and those who will not. Discrimination was assessed by the Concordance (c) statistic, the interpretation of which is equivalent to the area under the receiver operating characeristic (ROC) curve, that is, a c statistic of 0.5 indicates no discrimination above chance, whereas a c statistic of 1.0 indicates perfect discrimination. Comparison of c statistics between the model including pN Stage (Model A) with the one including LNR (Model B) tells whether one model is better in discriminating between poor and good survivors, and thus superior in predicting survival. Model calibration–the agreement between predicted risks and observed mortality risks–was assessed using the Hosmer Lemeshow test by comparing the predicted survival and the observed survival at 3-year follow-up.

### Net Reclassification Improvement of Patients Based on the Prediction Models

Finally, the c statistic has been criticized for being insensitive in comparing models and for having little direct clinical relevance. Therefore, we calculated the Net Reclassification Improvement (NRI), which assesses the ability of a model including a new prognostic marker (LNR - model B) to more accurately reclassify individuals into higher or lower risk(of death) category compared to model A, i.e., to check whether model B was better at correctly reclassifying patients into high risk and low risk groups based on their predicted survival probability as compared to model A. The NRI is the difference in proportions of patients moving up and down risk categories (high, moderate and low risk of mortality) among patients with the event of interest (death) versus those without (in our case patients who died within 3 years of follow up versus those who survived).The NRI is similar to the percentage reclassified but distinguishes between movements in the correct direction (patients moving up the risk categories for event patients (deaths) and down for nonevent patients (survivors)) [16]. Any upward movement in risk categories for subjects with the event (death) implies improved classification, and any downward movement indicates worse reclassification. The interpretation is opposite for subjects without the event (death).

The NRI is calculated as follows:

P_up,event_  =  number of events moving up/number of events.

P_down,event_  =  number of events moving down/number of events.

P_up,nonevent_  =  number of nonevents moving up/number of non events.

P_down,nonevent_  =  number of nonevents moving down/number of non events.

NRI  =  (P_up,event_ - P_down,event_) – (P_up,nonevent_ - P_down,nonevent_).

Where “up” refers to the patients moving up in the risk categories based on the new model when being compared to the old model and “down” refers to the patients moving down in the risk categories based on the new model when being compared to the old model.

In order to estimate P_up,event_, P_down,event,_ P_up,nonevent_, P_down,nonevent_, we first determined the the predicted survival probability for each patient based on models A and B. Based on this predicted survival probability patients were categorized into tertiles corresponding to low, intermediate and high risk of death at 3 years of follow up. The majority of the patients were correctly classified by both the models (as indicated by a high proportion of patients falling on the diagonals in the risk classification table).

After a recent publication suggested that LNR is particularly informative in subgroups of patients (i.e. patients with unfavorable tumor characteristics and younger patients) we performed subgroup analyses by age (<60 years and ≥60 years), receptor status (ER- vs ER+) and grade (1, 2 and 3) [10]. For each subgroup, two models were built as mentioned above.

All analyses were performed using STATA version 11.

## Results

According to the LNR classification, 758 (47.7%) patients were categorized as low category (>0 and <0.2), 574 (36.1%) as intermediate category (0.2 to 0.65) and 257 (16.2%) as high category (>0.65 to 1) LNR corresponding to low, intermediate and high risk of death respectively. For classic pN staging, 879 (55.2%) were pN1, 447 (28.1%) pN2 and 263 (16.7%) pN3 (Table 1). In all, 605 (38%) deaths were reported for the 1589 patients.

**Table 1:** Patient, tumor characteristics and treatment along with the unadjusted Hazard Ratio for all cause mortality.

Variable	N (%)	Unadjusted HR (95% CI)	P value of unadjusted HR
**Age in years**			<0.001
Median (Range)	50 (22 to 87)		
<40 years	225 (14.2%)	1	
40 to 49 years	569 (35.8%)	0.7 (0.5 to 0.9)	
50 to 59 years	470 (29.6%)	1.0 (0.8 to 1.3)	
≥60 years	325 (20.5%)	1.2 (0.9 to 1.6)	
**Year of diagnosis**			0.76
1990–2000	521 (32.8%)	1	
2001–2007	1068 (67.2%)	1.0 (0.8 to 1.2)	
**Place of Diagnosis**			<0.001
Kuala Lumpur	1015 (63.8)	1	
Singapore	574 (26.2%)	0.4 (0.3 to 0.5)	
**Ethnicity**			0.005
Chinese	1064 (67.0%)	1	
Malay	303 (19.1%)	1.2 (1.0 to 1.5)	
Indian	176 (11.1%)	1.5 (1.1 to 1.9)	
Other	46 (2.9%)	0.8 (0.4 to 1.6)	
**ER status** *			<0.001
Negative	662 (44.0%)	1	
Positive	844 (56.0%)	0.5 (0.4 to 0.7)	
Unknown	83	0.8 (0.5 to 1.1)	
**PR Status** *			<0.001
Negative	596 (45.7%)	1	
Positive	706 (54.3%)	0.4 (0.3 to 0.6)	
Unknown	287	0.8 (0.6 to 1.0)	
**Grade** *			<0.001
Low	89 (6.2%)	0.4 (0.2 to 0.7)	
Moderate	699 (49.1%)	1	
High	635 (44.6%)	1.4 (1.1 to 1.6)	
Unknown	166	1.0 (0.7 to 1.4)	
**Tumor size** *			<0.001
≤2 cm	381 (26.0%)	0.5 (0.4 to 0.7)	
2.1–5 cm	868 (59.3%)	1	
>5 cm	214 (14.6%)	1.6 (1.3 to 2.0)	
Unknown	126	0.8 (0.6 to 1.1)	
**Radiotherapy**			<0.001
No	430 (26.9%)	1	
Yes	1159 (72.9%)	0.7 (0.5 to 0.8)	
**Chemotherapy**			<0.001
No	246 (15.5%)	1	
Yes	1343 (84.5%)	0.5 (0.4 to 0.6)	
**Hormone Therapy**			<0.001
No	560 (35.2%)	1	
Yes	1029 (64.8%)	0.5 (0.4 to 0.6)	
**Regional nodes examined**			0.151
Median	15		
1–3	18 (1.1%)	1.8 (0.9 to 3.3)	
4–9	249 (15.7%)	1.0 (0.8 to 1.2)	
≥10	1322 (83.2%)	1	
**Regional nodes positive (pN Stage)**			<0.001
Median	3		
1–3	879 (55.2%)	1	
4–9	447 (28.1%)	1.7 (1.4 to 2.1)	
≥10	263 (16.7%)	3.3 (2.6 to 4.1)	
**Lymph Node Ratio**			<0.001
Median	0.22		
0.01–0.2	758 (47.7%)	1	
0.201–0.65	574 (36.1%)	1.5 (1.2 to 1.8)	
0.651–1	257 (16.2%)	3.6 (2.9 tp 4.5)	

* indicates valid proportions have been calculated (i.e., not considering unknown).

Five year survival probabilities for the patients categorized by LNR were 79%, 70% and 43% for low, intermediate and high category LNR groups respectively (Table 2). Five year survival probabilities for the patients categorized by pN classification were 79%, 65% and 48% for pN1, pN2 and pN3 respectively.

**Table 2:** Survival probabilities and Hazard Ratios for all cause mortality by pN classification and LNR.

Variable	N (%)	N of deaths	5 year Survival Probability (95% CI)	Unadjusted HR (95% CI)	Adjusted HR (95% CI)	c statistic (95% CI)
**pN Stage**						0.72 (0.70 to 0.75)
pN1	879 (55.2%)	256	79.0% (75.6% to 82.4%)	1	1	
pN2	447 (28.1%)	198	65.0% (59.0% to 71.0%)	1.7 (1.4 to 2.1)	1.9 (1.5 to 2.3)	
pN3	263 (16.7%)	151	48.0% (43.2% to 52.8%)	3.3 (2.6 to 4.1)	3.0 (2.4 to 3.7	
**Lymph Node Ratio**						0.73 (0.71 to 0.76)
Low ≤0.20	758 (47.7%)	213	79.0% (75.4% to 82.6%)	1	1	
Intermediate >0.20 to ≤0.65	574 (36.1%)	228	70.0% (65.2% to 74.8%)	1.5 (1.2 to 1.8)	1.5 (1.2 to 1.9)	
High >0.65	257 (16.2%)	164	43.0% (33.0% to 53.0%)	3.6 (2.9 to 4.5)	3.2 (2.6 to 4.0)	

* Model A is adjusted for: age, radiotherapy, surgery type, grade and tumor size and pN stage and stratified by ER Status. Model B is adjusted for: age, radiotherapy, surgery type, grade and tumor size and LNR and stratified by ER Status. Both models were internally validated using bootstrap resampling.

### Prediction Models

In univariate Cox regression analysis, age at diagnosis, place of diagnosis, year of diagnosis, ethnicity, receptor status (ER and PR), treatment, grade, stage, tumor size, pN staging were independently and significantly associated with all cause mortality (Table 1). After multivariate analysis, a model consisting of pN, age, tumor size, tumor grade, chemotherapy, radiotherapy, and surgery, gave the best fit. Taking pN1 patients as a reference, adjusted mortality risks (Hazard Ratios) were 1.9 (95%CI, 1.5 to 2.3) for pN2 patients and 3.0 (95%CI, 2.4 to 3.7) for pN3 patients. Similarly, compared to patient classified as low risk LNR (>0 and <0.2), those with intermediate risk LNR had an HRadj of 1.5 (95%CI, 1.2 to 1.9) and those with high risk LNR an HRadj of 3.2 (95%CI, 2.6 to 4.0) (Table 2).

### Discrimination and caliberation of prediction models

Both models A (base model plus pN) and B (base model plus LNR) were well calibrated (p-value Hosmer Lemeshow test 0.67 and 0.83 respectively). In terms of discriminating ability, both models performed equally well, as shown by the c statistic for model A of 0.72 (95% CI 0.70 to 0.75) and c statistic for the model B of 0.73 (95% CI 0.71 to 0.76). The substantial overlap between the two 95% confidence intervals indicated that LNR did not provide any added prognostic value when compared to pN staging in predicting all cause mortality.

### Net Reclassification Improvement of Patients Based on the Prediction Models

Based on individual predicted survival probabilities (from both pN staging and LNR models), when patients were categorized into tertiles of low, intermediate and high risk of death, the LNR model reclassified an additional 8.0% (n = 49) of patients with the event (death) into high risk groups and incorrectly reclassified 4.5% (n = 29) of the patients with the event into low risk groups. Among the patients without the event (alive), an additional 5.6% (n = 52) of patients were reclassified into low risk groups while 5.7% (n = 53) of the patients without the event were reclassified into high risk (Table 3).

**Table 3:** Risk reclassification table at 3 years of follow up based on models including pN stage and LNR respectively.

As per model A (with pN)					
		Low risk of death	Intermediate riskof death	High risk of death	Total
For patients withthe event (Dead)	Low risk of death	127	24		151
	Intermediate risk of death	23	335	25	383
	High risk of death		6	65	21
	Total	150	365	90	605
**As per model B (with LNR)**					
For patients without theevent (alive)	Low risk of death	405	45		450
	Intermediate risk of death	48	396	8	452
	High risk of death		4	16	20
	Total	453	445	24	922

Net Reclassification Index (NRI) = 3.2% (p value 0.08). Patients are categorized into risk categories of death based on their individual survival probabilities obtained from models A and B such that a patient with a high survival probability is categorized into the ‘low risk of death’ group and so on.

Subgroup analysis showed that LNR was superior to pN staging in categorizing patients’ risk of death for patients aged 60 years and above, patients with ER negative tumors and patients with high grade tumors, as in, for these subgroups, 95% confidence intervals (CIs) for intermediate and high risk LNR groups did not overlap while they did for the pN2 and pN3 categories. However, in terms of discriminating ability, models for all subgroup analyses including LNR performed as well as the models including pN respectively, as attested by the c statistics and largely overlapping 95% CIs (Table 4). There was no significant difference in between LNR and pN staging in terms of risk categorization for women aged less than 60 years, patients with ER positive tumors and patients with low and moderate grade tumors (Table S1).

**Table 4:** Subgroup analysis to check the added prognostic value of LNR over pN within specific subgroups.

Patients ≥60 years of age at diagnosis (N = 325)					
	N (%)	N Death (%)	Unadj HR (95% CI)	Adj HR^a^ (95% CI)	C statistic (95% CI)
**pN stage**					0.75 (0.70 to 0.81)
pN1	175 (53.8%)	53 (36.3%)	1	1	
pN2	89 (27.4%)	51 (34.9%)	2.8 (1.8 to 4.1)	2.7 (1.8 to 4.1)	
pN3	61 (18.8%)	42 (28.8%)	4.2 (2.7 to 6.3)	4.2 (2.6 to 6.7)	
**Lymph Node Ratio**					0.76 (0.71 to 0.80)
Low ≤0.20	147 (45.2%)	44 (30.1%)	1	1	
Intermediate >0.20 to ≤0.65	112 (34.5%)	51 (34.9%)	1.6 (1.0 to 2.4)	1.8 (1.1 to 2.7)	
High >0.65	66 (20.3)	51 (34.9%)	5.2 (3.4 to 7.8)	4.5 (2.8 to 7.0)	
**Patients with ER negative tumors at diagnosis (N = 662)**					
	**N (%)**	**N Death (%)**	**Unadj HR (95% CI)**	**Adj HR** b **(95% CI)**	**C statistic (95% CI)**
**pN stage**					0.84 (0.80 to 0.87)
pN1	339 (51.2%)	100 (36.0%)	1	1	
pN2	206 (31.1%)	106 (38.1%)	2.0 (1.5 to 2.6)	2.0 (1.5 to 2.7)	
pN3	117 (17.7%)	72 (25.9%)	3.1 (2.3 to 4.3)	3.0 (2.1 to 4.1)	
**Lymph Node Ratio**					0.85 (0.81 to 0.88)
Low ≤0.20	304 (45.9%)	93 (33.6%)	1	1	
Intermediate >0.20 to ≤0.65	233 (35.2%)	95 (33.9%)	1.4 (1.0 to 1.9)	1.5 (1.1 to 2.0)	
High >0.65	125 (18.9%)	90 (32.5%)	3.7 (2.7 to 4.9	3.5 (2.5 to 4.8)	
**Patients with high grade tumors at diagnosis (N = 635)**					
	**N (%)**	**N Death (%)**	**Unadj HR (95% CI)**	**Adj HR** c **(95% CI)**	**C statistic (95% CI)**
**pN stage**					0.76 (0.72 to 0.80)
pN1	320 (50.4%)	109 (40.1%)	1	1	
pN2	180 (28.3%)	84 (30.9%)	1.6 (1.2 to 2.1)	1.7 (1.2 to 2.3)	
pN3	135 (21.3%)	79 (29.0%)	2.6 (1.9 to 3.5)	2.6 (1.9 to 3.5)	
**Lymph Node Ratio**					0.76 (0.72 to 0.81)
Low ≤0.20	286 (45.0%)	100 (36.9%)	1	1	
Intermediate >0.20 to ≤0.65	229 (36.1%)	94 (34.7%)	1.3 (1.0 to 1.7)	1.4 (1.1 to 1.8)	
High >0.65	120 (18.9%)	77 (28.4%)	2.9 (2.1 to 3.1)	2.7 (2.0 to 3.7)	

a Model adjusted for age at diagnosis, chemotherapy, radiotherapy, surgery type, grade and tumor size and stratified by ER status.

b Model adjusted for age at diagnosis, chemotherapy, surgery type and tumor size.

c Model adjusted for age at diagnosis, chemotherapy, radiotherapy, surgery type and tumor size and stratified by ER status. All models were internally validated using bootstrap resampling.

Although a majority of the patients (∼83%) did have at least ten lymph nodes examined, about 17% of the patients had less than 10 nodes removed during axillary dissection. We performed a subgroup analysis to assess the added prognostic value of LNR for patients with less than 10 nodes retrieved but even for this subset of patients, both pN staging and LNR predicted all cause mortality equally well (data not shown). Different cut offs for LNR were tested for the entire dataset but no new cut offs of LNR for South East Asian patients were established.

## Discussion

This study shows that pN staging as well as the LNR are comparable in predicting overall survival of women with breast cancer, except for patients aged 60 or more, patients with ER negative tumors and patients with high grade tumors. Here, LNR was superior in categorizing patients into intermediate and high risk strata as compared to pN stage. However, in combination with other prognostic factors, LNR did not provide any additional prognostic information over pN staging, neither for the entire cohort, nor for the subgroups of older women and those with ER negative of grade 3 disease. The fact that LNR was not superior to the pN staging was seen in other Asian studies as well [10]. A non significant Net Reclassification Index for the LNR model compared to the pN model suggested that both LNR and pN stage were equally good at classifying patients into appropriate risk strata based on whether they experienced the event (death) or not.

There are several independent but interrelated prognostic factors that predict for recurrence and survival of breast cancer patients. These include amongst others, tumor size, axillary nodal status, histopathology, steroid receptors, HER 2 status, proliferative rate, ploidy, and oncogene amplification [17]. One of the strongest prognostic indicators for breast cancer is number of positive axillary nodes [18]. Furthermore, there is a direct relationship between the number of involved axillary nodes and the risk for distant recurrence [17].

The number of lymph nodes retrieved and examined is highly dependent on surgical expertise, the institution’s protocol and the pathologists’ experience [19]. Removal of at least ten axillary lymph nodes is considered adequate for reliable lymph node staging [20], [21], [22]. In the current study, 17% of the patients had less than 10 nodes removed during axillary dissection. Even for this subset of patients, both LNR and pN staging performed equally well in predicting all cause mortality and there was no significant difference in the discriminative power of the two multivariate models (one with LNR and one with pN).

Results from our study showed that LNR and pN were equally good at predicting all cause mortality overall but within certain subgroups (ER negative patients, patients aged 60 years or more and patients with high grade tumors), LNR was better at categorizing patients into risk categories. The intermediate category LNR was truly intermediate for these subgroups, i.e., the 95% Confidence Interval (CI) of the Hazard Ratio overlapped neither the low nor the high category LNRs, whereas the pN2 and pN3 CIs overlapped (Table 4). Thus in multivariate analyses, classification using the LNR provided well balanced nonoverlapping risk groups, whereas classification using pN provided poorly separated risk groups with overlapping hazard ratios for these subgroup of patients. However, when comparing the c statistics for the pN and LNR models for each of the subgroups respectively, there was no significant difference. This suggested that LNR did not provide any added prognostic value over pN stage for these subgroup of patients as well.

Recent studies have indicated that full axillary clearance following a positive sentinel node biopsy does not affect survival in certain (low risk) categories of breast cancer patients [23], [24]. These studies may induce a shift towards less axillary clearances following sentinel node biopsy in the future. However, in many low and middle income countries, sentinel node biopsies are not routinely available. Also, Asian women present with more advanced disease, larger tumor sizes, more nodal metastasis and more high grade tumors, and therefore complete axillary dissection is still very relevant in the South East Asian [14].

We acknowledge that our study suffers from several shortcomings, including a relatively short follow up time. In addition, we assessed all cause mortality as our end point as no data on cause of death was available. This could have led to a mixing of effects as this analysis allowed for competing risks of death. Also, additional information on HER2/NEU receptor status, socioecomonic status and comorbidity could have allowed for a deeper understanding of the association.

### Conclusion

Among South East Asian breast cancer patients, both the Lymph Node Ratio and the pN staging system seem to be equally good at predicting all cause mortality based on the cut offs used for LNR in this study. LNR may be better than pN in dividing tumors into high vs low risk for certain subgroup of patients, but LNR has no added prognostic value over pN staging in addition to other prognosticators.

## Supporting Information

Table S1 **Multivariate Cox regression analysis for all cause mortality by different subgroups.**
(DOCX)
