# Supplementary material for: Does the Axillary Lymph Node Ratio Have Any Added Prognostic Value over pN Staging for South East Asian Breast Cancer Patients?
Source: PLoS One. 2012 Sep 24;7(9):e45809. doi: 10.1371/journal.pone.0045809 (PMC3454359; doi:10.1371/journal.pone.0045809)
Supplement: Table S1 — Multivariate Cox regression analysis for all cause mortality by different subgroups. (DOCX) [file pone.0045809.s001.docx]

Supplementary Table 1: Multivariate Cox regression analysis for all cause mortality by different subgroups

| **Patients < 60 years of age at diagnosis (N=1264)** | | | | |
| --- | --- | --- | --- | --- |
|  | **N (%)** | **Unadj HR**  **(95% CI)** | **Adj HR^a^**  **(95% CI)** | **C statistic**  **(95% CI)** |
| **pN stage**  pN1  pN2  pN3 | 704 (55.7%)  358 (28.3%)  202 (16.0%) | 1  1.5 (1.2 to 1.9)  2.7 (2.1 to 3.4) | 1  1.6 (1.3 to 2.1)  2.6 (2.0 to 3.5) | 0.71 (0.68 to 0.74) |
| **Lymph Node Ratio**  Low ≤0.20  Intermediate >0.20 to ≤0.65  High >0.65 | 612 (48.5%)  462 (36.5%)  190 (15.0%) | 1  1.4 (1.1 to 1.7)  2.9 (2.2 to 3.6) | 1  1.6 (1.2 to 2.2)  2.9 (2.2 to 3.9) | 0.70 (0.66 to 0.72) |
| **Patients with ER positive tumors at diagnosis (N=844)** | | | | |
|  | **N (%)** | **Unadj HR**  **(95% CI)** | **Adj HR^b^**  **(95% CI)** | **C statistic**  **(95% CI)** |
| **pN stage**  pN1  pN2  pN3 | 485 (57.5%)  222 (26.3%)  137 (16.2%) | 1  1.5 (1.1 to 2.1)  2.9 (2.1 to 3.8) | 1  1.6 (1.2 to 2.3)  2.9 (2.1 to 4.1) | 0.74 (0.71 to 0.79) |
| **Lymph Node Ratio**  Low ≤0.20  Intermediate >0.20 to ≤0.65  High >0.65 | 423 (50.1%)  307 (36.4%)  114 (13.5%) | 1  1.6 (1.2 to 2.0)  2.6 (1.9 to 3.6) | 1  1.8 (1.3 to 3.4)  2.9 (2.0 to 4.2) | 0.75 (0.70 to 0.77) |
| **Patients with low grade tumors at diagnosis (N=89)** | | | | |
|  | **N (%)** | **Unadj HR**  **(95% CI)** | **Adj HR^c^**  **(95% CI)** | **C statistic**  **(95% CI)** |
| **pN stage**  pN1  pN2  pN3 | 61 (68.5%)  21 (23.6%)  7 (7.9%) | 1  3.1 (1.1 to 8.1)  17.4 (4.3 to 69.0) | 1  2.9 (0.9 to 9.5)  30.3 (5.4 to 171.7) | 0.68 (0.64 to 0.78) |
| **Lymph Node Ratio**  Low ≤0.20  Intermediate >0.20 to ≤0.65  High >0.65 | 53 (59.6%)  28 (31.4%)  8 (9.0%) | 1  2.0 (1.0 to 5.0)  2.8 (0.7 to 10.7) | 1  2.9 (1.0 to 8.9)  5.2 (1.0 to 25.4) | 0.67 (0.64 to 0.79) |
| **Patients with moderate grade tumors at diagnosis (N=699)** | | | | |
|  | **N (%)** | **Unadj HR**  **(95% CI)** | **Adj HR^c^**  **(95% CI)** | **C statistic**  **(95% CI)** |
| **pN stage**  pN1  pN2  pN3 | 402 (57.5%)  192 (27.5%)  105 (15.0%) | 1  1.8 (1.3 to 2.4)  2.8 (2.0 to 3.9) | 1  2.0 (1.4 to 2.7)  3.0 (2.1 to 4.3) | 0.70 (0.66 to 0.74) |
| **Lymph Node Ratio**  Low ≤0.20  Intermediate >0.20 to ≤0.65  High >0.65 | 347 (49.6%)  253 (46.2%)  99 (14.2%) | 1  1.7 (1.3 to 2.3)  3.3 (2.4 to 4.7) | 1  1.9 (1.4 to 2.6)  3.4 (2.3 to 5.0) | 0.71 (0.67 to 0.74) |
| ^a^ Model adjusted for age at diagnosis, chemotherapy, radiotherapy, surgery type, grade and tumor size and stratified by ER status  ^b^ Model adjusted for age at diagnosis, chemotherapy, , surgery type and tumor size  ^c^ Model adjusted for age at diagnosis, chemotherapy, radiotherapy, surgery type and tumor size and stratified by ER status  All models were internally validated using bootstrap resampling. | | | | |
